# The Role of γδ T Cells in Fibrotic Diseases

**DOI:** 10.5041/RMMJ.10256

**Published:** 2016-10-31

**Authors:** Ilan Bank

**Affiliations:** Department of Medicine, Maayenei Hayeshuah Medical Center, Bnei Brak, Israel; Rheumatology Unit, Autoimmunity Institute and Laboratory of Immunoregulation, Sheba Medical Center, Ramat Gan, Israel; and Sackler School of Medicine, Tel Aviv University, Tel Aviv, Israel

**Keywords:** Fibrosis, systemic sclerosis, γδ T cells

## Abstract

Inflammation induced by toxins, micro-organisms, or autoimmunity may result in pathogenic fibrosis, leading to long-term tissue dysfunction, morbidity, and mortality. Immune cells play a role in both induction and resolution of fibrosis. γδ T cells are an important group of unconventional T cells characterized by their expression of non-major histocompatibility complex restricted clonotypic T cell receptors for non-peptide antigens. Accumulating evidence suggests that subsets of γδ T cells in experimentally induced fibrosis following bleomycin treatment, or infection with *Bacillus subtilis*, play pro-inflammatory roles that instigate fibrosis, whereas the same cells may also play a role in resolving fibrosis. These processes appear to be linked at least in part to the cytokines produced by the cells at various stages, with interleukin (IL)-17 playing a central role in the inflammatory phase driving fibrosis, but later secretion of IL-22, interferon γ, and CXCL10 preventing pathologic fibrosis. Moreover, γδ T cells appear to be involved, in an antigen-driven manner, in the prototypic human fibrotic disease, systemic sclerosis (SSc). In this paper we review in brief the scientific publications that have implicated γδ T cells in fibrotic diseases and their pro- and anti-fibrotic effects.

## INTRODUCTION

Extensive tissue deposition of extracellular matrix proteins by activated fibroblasts may lead to structural and functional tissue damage. Uncontrolled fibrosis may be a consequence of inflammation triggered by pathogens, autoimmunity or malignancies, and is related to dysregulation of multiple types of immune cells including subsets of T cells.^1^ γδ T cells, a “non-conventional” T cell population, were discovered in 1986 and, in contrast to “conventional” T cells expressing the αβ T cell receptor (TCR), recognize non-peptidic antigens independent of major histocompatibility (MHC) molecules.^2^–^5^ In humans there are two major subsets; the first expresses TCR γδ That use variable (V) region genes Vγ9 and Vδ2 in the γ and δ TCR polypeptides, respectively. Vγ9δ2 TCR sense low-molecular-weight phosphoantigens of microbes, and host cell-produced phosphoantigens in the mevalonate pathway.^6^ These phosphoantigens bind to the extra- or intracellular domains of the cell surface membrane molecule butyrophilin 3A1 (CD277), inducing a novel structure or conformation that is detected by cells expressing the Vγ9δ2 TCR, triggering their cytokine production and/or cytotoxicity.^7^,^8^ Thus, Vγ9δ2 γδ T cells are poised to detect and respond to infections or altered intracellular metabolism induced, for example, by intracellular infections, or a malignant transformation. The second human γδ T cell subset is characterized by the Vδ1 genes in the δ TCR polypeptide. Vδ1^+^ γδ T cells are distributed along epithelial barriers. Their TCR detects lipid antigens presented by CD1 molecules, similar to natural killer T (NKT) cells.^9^,^10^ Although the murine immune system lacks phosphoantigen-reactive γδ T cells, the role of butyrophilins in γδ T cell development is retained in mice, at least for some subsets, as exemplified by the dependence of entire subsets of murine γδ T cells on specific butyrophilins for their development and homing to the skin and gut.^11^–^13^ Readers are referred to comprehensive reviews of murine γδ T cells by Vantourout and Hayday.^4^,^5^

Despite obvious distinctions between the murine and human γδ T cells, there is ample evidence to indicate that the functional repertoire of γδ T cells in both humans and mice includes cytokine production, cytotoxicity, and help for B cells.^4^ Uniquely, moreover, subsets of these cells acquire their full functional potential during maturation in the thymus, contrasting with αβ T cells that fully mature functionally only after encountering antigens in the peripheral lymphatic system. In this regard, in both humans and mice, γδ T cells are similar to innate lymphocytes, which positions them at the forefront of the response to foreign invaders and internal “stress” conditions, including, for example, metabolic aberrations induced by malignancy, infections or other stressogens.^4^ Indeed, inflammatory, malignant, and infectious conditions are associated with numerical alterations of γδ T cells in humans.^14^ Given their unique abilities to detect non-peptide antigens, that may evade adaptive αβ T cells, and their rapid, non-MHC-dependent responsiveness, these cells may thus play a critical and unique role in diseases. Here, we review the involvement of the γδ T cell subset in pathological fibrotic responses. Specifically, we concentrate on systemic sclerosis (SSc), the prototypic systemic fibrosing disease in humans, and on animal models in experimental settings mimicking SSc, as well as in organ-localized pulmonary and liver fibrosis.

## γδ T CELLS IN HUMAN FIBROSIS

Most of the evidence linking human γδ T cells to fibrosis comes from studies of the systemic sclerosis (SSc). Thus, in SSc, Vδ1^+^ γδ T cells were identified in the skin during very early stages of SSc.^15^ Furthermore, the diversity of Vδ1 junctional regions (composed of the variable [V], diversity [D], and joining [J] gene segments) in peripheral blood (PB) mononuclear cells (PBMC), lung, esophagus, stomach, or skin of patients was limited in SSc patients, and the same Vδ1-Jδ junctional sequences could be isolated from multiple tissues suggesting an antigen-driven expansion of Vδ1^+^ γδ T cells in SSc.^16^ In a large group of patients, percentages of PB γδ T cells were significantly lower in SSc patients with diffuse and late-stage disease with pulmonary involvement, muscle involvement, and the presence of anti-Scl-70 antibodies, mimicking the University of California at Davis line (UCD)-200 chicken model described below.^17^ In addition, Vγ9^+^ γδ T cells persist in SSc patients’ PB, respond by expression of CD25 and CD69 to a phosphoantigen, isopentenyl pyrophosphate (IPP), and induce contact-dependent, tumor necrosis factor (TNF) α-independent apoptosis of cultured synovial fibroblasts.^18^ However, higher concentrations of zoledronate, an aminobisphosphonate that increases IPP by inhibiting intracellular farnesyl pyrophosphate (FPP) synthase, were required for maximal proliferation of Vγ9^+^ T cells in SSc patients than in healthy controls, suggesting their dysfunction in SSc; yet these cells still secreted factors that inhibited collagen production.^19^ Furthermore, less anti-fibrotic cytokines TNF-α and IFN-γ were secreted in response to IPP in SSc. Indeed, reduction of procollagen secretion by fibroblasts cultured with supernatants of IPP-stimulated PBMC was observed only in some SSc patients.^19^ On the other hand, γδ T cell supernatants from patients induced more proliferation of fibroblasts than αβ T cell supernatants, and doubling of collagen synthesis in human skin fibroblasts maintained in supernatants of SSc-derived γδ T cells was observed, which was inhibited by anti-transforming growth factor-beta (TGFβ) antibody and anti-basic-fibroblast growth factor antibodies.^20^ Furthermore, PB γδ T cells of SSc patients expressed higher levels of CD16 and CD69 compared to healthy controls, and collagen gene 1 (COL1) A2 mRNA expression was significantly higher in fibroblasts co-cultured with γδ T cells from SSc patients.^21^

## ANIMAL MODELS

### Systemic Sclerosis

The first indication that γδ T cells participate in the pathogenesis of fibrotic conditions arose from research in UCD-200 chickens. These animals develop a hereditary connective tissue disease characterized by severe lymphocytic infiltration and fibrosis of skin and internal organs, a model of human progressive SSc. The skin infiltrating mononuclear cells in the deeper dermis were mainly TCR αβ cells, whereas the perivascular area of the papillary dermis was enriched for TCR γδ^+^ lymphocytes.^22^

### Pulmonary Fibrosis Induced by a Non-Infectious Trigger

#### Bleomycin model: evidence for involvement of γδ T cells

In the bleomycin (BLM) model of lung fibrosis induced by a single intratracheal instillation of BLM, >80% of the γδ T cells in bronchoalveolar lavage (BAL) fluid expressed the E-cadherin binding αEβ7 integrin, at levels that were 2–3 times higher than on CD4^+^ or CD8^+^ T cells, suggesting a critical role for γδ T cells in the pathogenesis of BLM-induced lung fibrosis.^23^ After exposure to BLM, but not to *Schistosoma mansoni* eggs, the interleukin (IL)-17A that was produced by CD4^+^ and γδ T cells induced significant neutrophilia and pulmonary fibrosis. In parallel, IL-17A and IL-1β were increased in the BAL fluid of patients with idiopathic pulmonary fibrosis (IPF).^24^ Bleomycin or IL-1β-induced lung injury also led to increased expression of early IL-23p19 and IL-17A or IL-17F. A very early IL-17A and IL-17F expression by ROR γt(+) γδ T cells could be demonstrated 24 h after BLM administration. In addition, IL-23p19 and IL-17A expressions or IL-17RA signaling were necessary for pulmonary TGFβ1 production, collagen deposition, and evolution to fibrosis.^25^ Likewise, in the surfactant protein C/TNFα (SP-C/TNF) transgenic mouse, where the TNFα transgene is overexpressed in type II pneumocytes, the absolute number of lymphocytes recovered were approximately four times that in littermates, and included γδ T cells and B1 cells. In these mice the pulmonary lymphocytic infiltration is followed by fibrotic changes including accumulation of fibroblasts and deposition of extracellular matrix.^26^ Moreover, when experimental animals were injected intravenously with saline or collagen (Col)V 10 days before intratracheal instillation of BLM, ColV-pretreated animals showed a significant reduction in lung inflammation compared with non-treated animals which associated with a lower proportion of γδ and CD4^+^ T cells.^27^ After lung injury by BLM, γδ T cells localized to the lung lesions and were the predominant source of IL-17 by flow cytometry and real-time polymerase chain reaction (PCR). γδ T cell knockout (KO) mice showed a significant reduction in cellular infiltration into the airways, reduced expression of IL-6 in the lung, a significant delay in epithelial repair, and increased inflammation and fibrosis.^28^ In another study, although γδ T cell populations increased after BLM administration, pulmonary fibrosis was more severe in γδ KO mice, as measured by collagen deposition (hydroxyproline) and histopathological features. Furthermore, there was no evidence of resolution of the fibrotic response up to 45 days after BLM therapy. γδ KO mice had decreased concentrations of IL-6, granulocyte colony-stimulating factor, chemokine C-X-C ligand (CXCL) 1, and interferon-inducible protein 10/CXCL10. Importantly, γδ T cells produced all four of these cytokines, and γδ T cells sorted from BLM-treated lung were sufficient to resolve fibrosis in γδ KO mice. Overexpression of CXCL10 in the lung decreased the severity of fibrosis seen in the γδ KO mice, and adoptive transfer of γδ T cells from CXCL10(−/−) mice failed to reverse the severe fibrosis in γδ KO mice. Thus, γδ T cells promote resolution of fibrosis through production of CXCL10.^29^ In addition, BLM-treated mice showed decreased levels of IL-22 in the lung, and IL-22-producing γδ T cells were also decreased significantly in the lungs and spleens. Blockade of IL-22 deteriorated pulmonary fibrosis, and was associated with elevated α-smooth muscle actin and overactivated Smad2. Thus, IL-22 produced by γδ T cells may play a protective role in BLM-induced pulmonary fibrosis.^30^ Furthermore, BLM-induced lung inflammation and subsequent fibrosis was ameliorated in osteopontin (OPN)-deficient mice, whereas OPN was expressed ubiquitously in the lung parenchymal and bone marrow-derived components. The TH17 differentiation of CD4^+^ αβ T cells and IL-17-producing γδ T cells was reduced in OPN-deficient mice compared to wild-type mice, whereas TH1 differentiation and the percentage of IFN-γ-producing γδ T cells increased. Thus, OPN expressed in both parenchymal and bone marrow cell components contributed to BLM-induced lung inflammation and fibrosis by affecting the ratio of pathogenic IL-17/protective IFN-γ T cells.^31^

#### Silicosis model

Silicosis evolved over months after exposure of inbred mice to cristobalite silica with accumulation of lymphocytes in alveolar spaces, in lung parenchymal lesions and nodules, and in enlarged bronchial-associated lymphoid tissues and thoracic lymph nodes. The lung lymphocytes were predominantly CD4^+^ T cells, with numerous CD8^+^ T cells, natural killer cells, and γδ T cells.^32^ In another study upregulation of IL-17A was associated with the development of experimental silicosis, but was markedly reduced in athymic, γδ T cell-deficient or CD4^+^ T cell-depleted mice. γδ T lymphocytes and CD4^+^ T cells, but not macrophages, neutrophils, NK cells, or CD8 T cells, purified from the lungs of silicotic mice, markedly expressed IL-17A. Acute alveolitis induced by silica was IL-17A-dependent, but was dispensable for the late inflammatory and fibrotic lung responses.^33^

#### Melphalan model

Exposure to melphalan, a nitrogen mustard, induced an early burst of the pro-inflammatory cytokines IL-1β, IL-6, and IL-23 in airways, followed by extensive infiltration of neutrophils in the lung tissue and airways. The acute phase was followed by a sustained lymphocytic response that persisted for at least 14 days with resulting lung fibrosis. Engagement of T lymphocytes, particularly the γδ T cell subset, was crucial both for the acute cytokine and neutrophil response and for the late-phase lung fibrosis as indicated by the lack of response in γδ T cell-deficient mice.^34^

### Pulmonary Fibrosis Following a Bacterial Infection

#### Bacillus subtilis

C57BL/6 mice repeatedly exposed to *Bacillus subtilis* develop mononuclear infiltrates containing Vγ6^+^/Vδ1^+^ γδ T cells in the lung. In the absence of these, mice treated with *B. subtilis* had significantly increased collagen deposition in the lung, consistent with a regulatory role for Vγ6^+^/Vδ1^+^ γδ T cells. Exposing transgenic Vγ6^+^/Vδ1^+^ mice to *B. subtilis* decreased collagen content in the lung compared with wild-type C57BL/6 mice. Cytokine analysis of lungs from wild-type mice repeatedly exposed to *B. subtilis* demonstrated increased IL-17A concentrations. In the absence of IL-17 receptor signaling, IL-17ra(−/−) mice had delayed clearance of *B. subtilis*, with increased lung inflammation and fibrosis. Although IL-17A was predominantly expressed by Vγ6^+^/Vδ1^+^ γδ T cells, a compensatory increase in IL-17A expression by CD4^+^ T cells was seen in the absence of γδ T cells that resulted in similar levels of IL-17A in the lungs of TCRδ(−/−) and wild-type C57BL/6 mice, suggesting an important role for IL-17A-expressing γδ or αβ T lymphocytes in eliminating the micro-organism and preventing excessive inflammation and eventual lung fibrosis.^35^ Likewise, in another study of this mouse model, γδ T cells expanded in the lung and inhibited collagen deposition. A subset of these γδ cells represents the predominant source of the TH17 cytokine IL-22 in this model. Preventing expression of IL-22 by mutating the aryl hydrocarbon receptor (AhR)—or inhibiting AhR signaling—accelerated lung fibrosis. Moreover, the presence of protective γδ T cells and IL-22 diminished recruitment of CD4^+^ T cells to lung.^36^ Finally, repeatedly exposing C57BL/6 mice to *B. subtilis* resulted in a 33-fold increase in the number of CD4^+^ T cells and a 354-fold increase in γδ T cells in the lung. The γδ T cells consisted almost entirely of Vγ6^+^/Vδ1^+^ γδ T cells. Treatment of C57BL/6 mice with heat-killed versus live *B. subtilis* resulted in a 2-fold increase in the number of CD4^+^ T cells in the lung but no expansion of γδ T cells. In addition, mice treated with heat-killed *B. subtilis* developed significantly increased pulmonary fibrosis compared with mice treated with the live micro-organism. Mice deficient in Vγ6^+^/Vδ1^+^ γδ T cells, when treated with *B. subtilis*, had a 231-fold increase in lung CD4+ T cells and significantly increased collagen deposition compared with wild-type C57BL/6 mice, again consistent with an immunoregulatory role for the Vγ6^+^/Vδ1^+^ γδ T cell subset.^35^

#### Tuberculosis

The acute phase of pulmonary tuberculosis induced in BALB/c mice by the intratracheal instillation of the live virulent strain H-37Rv was characterized by an inflammatory infiltrate in the alveolar capillary interstitium, blood vessel, and bronchial wall with formation of granulomas from 1 to 28 days after infection and a predominance of TH1 cells. The chronic phase was characterized by pneumonia, focal necrosis, and fibrosis. γδ T lymphocytes were involved both at the beginning (3 days) and the later stages of the infection.^37^ In bovine tuberculosis, there was an increase in the expression of TGFβ, and of type I procollagen in advanced stage granulomas. As the granulomas advanced, there was a steady increase in the number of CD68^+^ cells and γδ T cells.^38^

### Liver Fibrosis Induced by a Non-Infectious Trigger

#### Carbon tetrachloride model

Increased IL-17A production was mainly detected in hepatic γδ T cells in wild-type mice. Liver fibrosis and IL-17A production by γδ T cells were both significantly attenuated in toll-like receptor (TLR)-3 KO mice compared with wild-type mice. Interleukin-17A-producing γδ T cells were in close contact with activated hepatic stellate cells (HSCs), suggesting a role for HSCs in IL-17A production by γδ T cells. Interleukin-17A production by γδ T cells was substantially increased upon co-culturing with exosome-treated wild-type HSCs or conditioned medium from TLR3-activated wild-type HSCs. Toll-like receptor-3 deficiency in HSCs contributed to decreased IL-17A production by γδ T cells, as well as liver fibrosis. Thus, in liver injury, the exosome-mediated activation of TLR3 in HSCs exacerbates liver fibrosis by enhancing IL-17A production by γδ T cells, which might be associated with HSC stimulation by unknown self-TLR3 ligands from damaged hepatocytes.^39^ Chemokine receptor 6 (CCR6) and chemokine ligand (CCL) 20 expression were intrahepatically upregulated in patients with chronic liver diseases compared to control liver, with periportal accumulation of CCR6(+) mononuclear cells and CCL20 induction by hepatic parenchymal cells. In murine livers CCR6 was expressed by macrophages, CD4^+^, and γδ T cells and upregulated in fibrosis, whereas CCL20 was induced by injury in primary hepatocytes. In the carbon tetrachloride (CCl_4_) and methionine-choline-deficient diet-induced murine models of chronic liver injury, Ccr6(−/−) mice developed more severe fibrosis with enhanced immune cell infiltration than wild-type mice, and CCR6 was required by IL-17- and IL-22-expressing γδ T cells for accumulation in injured liver. Adoptive transfer of wild-type γδ, but not CD4^+^ T cells, into Ccr6(−/−) mice reduced hepatic inflammation and fibrosis in chronic injury to wild-type level. The anti-inflammatory function of hepatic γδ T cells was independent of IL-17, whereas γδ T cells co-localized with HSCs *in vivo* and promoted apoptosis of primary murine HSCs in a cell–cell contact-dependent manner, involving Fas-ligand (CD95L).^40^

### Liver Fibrosis Induced by an Infectious Agent

#### Fasciola hepatica (fluke)

Ten days after primary infection with *Fasciola hepatica* (fluke), portal tract areas surrounding migratory tunnels were infiltrated with T cells and B cells. Micro-abscesses were distributed sporadically in the liver parenchyma, and young flukes were observed in the liver tissue free from inflammatory cells. Chronic primary infections were characterized by perilobular fibrosis and a predominance of CD8^+^ and γδ T cells.^41^

#### Cryptosporidium parvum

Inoculation of mice deficient in αβ and γδ T cells with *Cryptosporidium parvum* resulted in persistent infection and severe inflammatory bowel disease-like lesions contrasting with neonatal immunocompetent strains of mice which results in a transient, non-inflammatory enteric infection. Glandular hyperplasia, abscess formation, and extensive fibrosis of the lamina propria and extensive hepatic periportal fibrosis were noted in persistently infected mice, which were not observed in mice deficient only in αβ T cells.^42^

#### Rotavirus

Livers from rhesus rotavirus-infected mice that develop biliary atresia (BA) had 7-fold more IL-17 messenger RNA than control mice (*P*=0.02). γδ T cells were the exclusive source of IL-17. Mice that were developing BA and given antibodies against IL-17 had lower levels of liver inflammation. Likewise, liver tissues from patients with BA had 4.6-fold higher levels of IL-17 messenger RNA than control liver tissues (*P*=0.02).^43^

#### Schistosoma japonicum

In C57BL/6 mice infected with *S. japonicum* expression and release of IL-17 was significantly higher in hepatic lymphocytes from infected mice. Interleukin-17 was induced in all CD4^+^ and NK cells by PMA and ionomycin, but γδ T lymphocytes exhibited the largest increase. Reducing IL-17 activity using anti-IL-17A antibodies decreased infiltration of inflammatory cells and collagen deposition in the livers of infected C57BL/6 mice.^44^

## CONCLUSION

**Figure 1:**
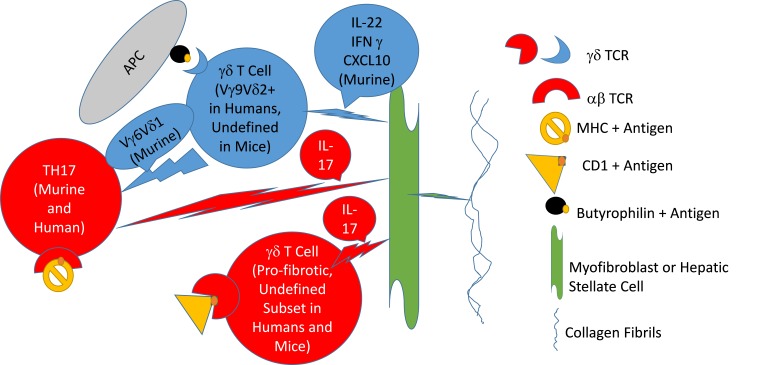
Hypothetical Model of γδ T Cell Involvement in Fibrosis A hypothetical model is depicted of how two types of γδ T cells, a T helper (TH) cell antigen-presenting cell (APC) and a myofibroblast, are involved in induction collagen secretion. The APCs are depicted presenting a peptidic antigen in MHC to the TH17 αβ T cell receptor, or a lipid antigen to a γδ T cell via a CD1 molecule, eliciting release of IL-17 that activates the myofibroblast to secrete collagen. Other γδ T cells, of the phosphoantigen-recognizing variety in humans, or, in the murine system, a subset secreting IL-22 and CXCL10, may become activated by other antigens presented by butyrophilins, to exert anti-fibrotic activity by inducing apoptosis of the myofibroblast or hepatic stellate cells, or by suppressing TH17 cells. Red depicts pro- and blue anti-fibrotic functions.

In summary, the data clearly indicate the involvement of γδ T cells in major human fibrotic diseases, as well as in models of post-inflammatory fibrosis in animals. The experimental models, however, suggest dual involvement: a role in induction of inflammation that can lead to fibrosis by IL-17-secreting γδ T cells, contrasting with a role in prevention of fibrosis related to γδ T cells that mediate either killing of cells responsible for secreting the extracellular matrix, or by subsets of these cells that secrete either matrix-degrading enzymes, IL-22, CXCL10, or IFNγ (Table 1, Figure 1, and Workalemahu et al.^45^). Much further study is required to elucidate the mechanisms that control pro- and anti-fibrotic effects of γδ T cells in human disease, since manipulation of these responses might enable prevention or alleviation of severe human fibrotic diseases.

**Table 1:** Models and Mechanisms of Pro- and Anti-fibrotic Effects of γδ T Cells**.**

Model	Pro-fibrotic	Anti-fibrotic	Mechanism	Ref
BLM-induced murine lung fibrosis	+		IL-17 production by γδ T and TH17 cells	25
BLM-induced murine lung fibrosis		+	Production of CXCL10 by γδ T cells	29
BLM-induced murine lung fibrosis		+	IL-22 produced by γδ T cells	30
BLM-induced lung fibrosis in osteopontin-deficient mice		+	IFN-γ-producing γδ T cells	31
Melphalan-induced murine lung fibrosis	+		Induction of pro-inflammatory cytokines, e.g. IL-6 and IL-1β	34
*Bacillus subtilis*-induced murine lung fibrosis		+	IL-17A-expressing γδ T cells involvement in removal of offending organism	35
*Bacillus subtilis*-induced murine lung fibrosis		+	Production of IL-22 by γδ T cells	36
*Bacillus subtilis*-induced murine lung fibrosis		+	Immunoregulatory role of Vγ6/Vδ1(+) γδ T cell subset	35
Carbon tetrachloride (CCl_4_) murine model of liver fibrosis	+		TLR3 activation of IL-17 secretion by γδ T cells	39
Carbon tetrachloride (CCl_4_) murine model of liver fibrosis		+	Promotion of apoptosis of hepatic stellate cells by γδ T cells	40
*Cryptosporidium parvum* infection-induced murine liver periportal fibrosis	+		No mechanism presented	42
Rotavirus infection inducing murine biliary atresia	+		IL-17 production by γδ T cells	43
*Schistosoma japonicum*-induced murine liver fibrosis	+		IL-17 production by γδ T cells	44
*In vitro* experiments using human cells		+	Cell contact-dependent apoptosis of fibroblasts and reduction of collagen secretion byproducts of Vγ9Vδ2+T cells	18, 19
*In vitro* human experiments	+		Increased fibroblast proliferation and collagen production by supernatants of γδ T cells of systemic sclerosis patients	20, 21

BLM, Bleomycin; CXCL10, C-X-C motif chemokine 10; IL, interleukin; TH, T helper; TL, toll-like.

## Abbreviations

AhR: aryl hydrocarbon receptor
BAL: bronchoalveolar lavage
BLM: bleomycin
CCL: chemokine ligand
CXCL: chemokine C-X-C ligand
FPP: farnesyl pyrophosphate
IL: interleukin
IPP: isopentenyl pyrophosphate
KO: knockout
MHC: major histocompatibility
NKT: natural killer T
PBMC: peripheral blood mononuclear cells
r: receptor
SSc: systemic sclerosis
TCR: T cell receptor
UCD-200: University of California at Davis line 200
V: variable
